# HPV Status and Its Correlation with BCL2, p21, p53, Rb, and Survivin Expression in Breast Cancer in a Chinese Population

**DOI:** 10.1155/2017/6315392

**Published:** 2017-12-20

**Authors:** Ya-Wen Wang, Kai Zhang, Song Zhao, Yanrong Lv, Jiang Zhu, Huantao Liu, Jinbo Feng, Weili Liang, Rong Ma, Jianli Wang

**Affiliations:** ^1^Department of Breast Surgery, Qilu Hospital of Shandong University, Jinan, Shandong, China; ^2^Gene Laboratory, Department of Obstetrics and Gynecology, Qilu Hospital of Shandong University, Jinan, Shandong, China; ^3^Department of Pathophysiology, School of Medicine, Shandong University, Jinan, Shandong, China

## Abstract

Despite recent evidence, the role of human papillomavirus (HPV) in breast carcinogenesis is controversial. The correlations of HPV infection with the clinicopathological features of breast cancer and the expression of cell cycle/apoptosis-associated proteins have not been well elucidated. In this study, we sought to determine the prevalence of high-risk HPVs (HR-HPVs) infection and BCL2, p21, p53, Rb, and survivin expression in breast cancer patients and to investigate the relationship of HPV with these cancer-related proteins, in an attempt to clarify the potential mechanism of HPV in breast cancer pathogenesis. HPV presence in 81 fresh breast cancer tissues was determined by hybrid capture 2 (HC2) assay, and expression of BCL2, p21, p53, Rb, and survivin was detected by immunohistochemistry. Here we showed that fourteen (17.3%) patients were HR-HPV positive. HPV infection demonstrated no significant correlation with the clinicopathological characteristics of breast cancer. HPV-positive tumors showed significantly higher BCL2 and lower p53 expression than HPV-negative tumors. Expression of p21, Rb, and survivin was not associated with HPV status. Our results suggest a possible role of HR-HPV in breast cancer carcinogenesis, in which BCL2 and p53 may be involved.

## 1. Introduction

Recent studies have reported that some viruses such as Epstein-Barr virus (EBV) and mouse mammary tumor virus (MMTV), as well as human papillomavirus (HPV), may play important roles in breast cancer development and progression [1]. The relationship between HPV and other types of cancers, including cervix, vagina, vulva, head and neck, anal, and penile carcinomas, has been well established [2]. However, reports on the association between HPV and breast cancer were controversial. The prevalence of HPV in breast cancer tissues ranged from 0 to 86% [3]. Although a number of studies have supported the involvement of HPV in breast cancer, several other investigations did not detect any HPV subtypes in breast cancer tissues [4]. It is important to further clarify the role and mechanism of HPV in breast cancer.

HPVs are small, circular, double-stranded DNA viruses. Approximately 200 different HPVs have now been identified and these viruses can be classified into mucosal and cutaneous HPVs [5]. The mucosal HPV types are designated as “low-risk” and “high-risk” types based on the propensity for malignant progression of the lesions that they cause [5]. Low-risk HPV subtypes, such as HPV 6 and HPV 11, cause more than 90% of genital warts, whereas high-risk HPV subtypes (HPVs 16, 18, 31, 33, 35, 39, 45, 51, 52, 56, 58, 59, and 68) cause squamous intraepithelial lesions that can progress to invasive squamous cell carcinomas [4]. Cell cycle and apoptosis are critical events during cell transformation and carcinogenesis [6]. The high-risk HPV E6 and E7 oncoproteins, which are consistently expressed in cancer, can inactivate the p53 and Rb tumor suppressors, respectively [7]. P53 and Rb are well-known apoptosis regulators that inhibit cell cycle progression and induce cellular growth arrest and apoptosis [8]. P53 might repress the transcription of the apoptosis regulator BCL2 and promote the expression of p21, a member of cyclin-dependent kinases (CDKs) inhibitor family (CDKI). The antiapoptosis gene survivin, expressed in cancer and lymphoma, has attracted research attention through the last decade [9]. Although several studies have demonstrated the relationship between HPV and BCL2, p21, p53, Rb, and survivin in malignant and premalignant lesions of uterine cervix [10], vulvar carcinoma [11], and oral carcinoma [12], little is known about the effect of HPV on these cell cycle/apoptosis-associated proteins in breast cancer.

In this study, we aim to determine the prevalence of HPV in tumors from breast cancer patients and to analyze its correlation with clinicopathological characteristics. Importantly, we also explored whether expressions of tumor suppressors p21, p53, and Rb and antiapoptosis proteins BCL2 and survivin were associated with HPV infection in breast cancer.

## 2. Materials and Methods

### 2.1. Sample Collection

Eighty-one fresh breast cancer samples were collected from Qilu Hospital of Shandong University (Jinan, China) between March 2012 and August 2012. All samples were confirmed by histopathological diagnosis. None of the patients included in this study received any adjuvant chemotherapy or radiotherapy prior to the operation. This study was approved by the Ethics Committee of Qilu Hospital of Shandong University (Jinan, China). Written informed consent was obtained from all the patients. Tumor cell specimens for hybrid capture 2 (HC2) testing were collected and stored as previously described [13].

### 2.2. HR-HPV HC2 Assay

HC2 testing was performed using the HC2 High-Risk HPV DNA Test kit (Digene, Gaithersburg, MD) according to the manufacturer's instructions to detect the presence of high-risk HPV. The HC2 assay used in vitro nucleic acid hybridization for qualitative detection of 13 subtypes of high-risk HPV. However, specific HPV types could not be determined. Target DNA in specimens was hybridized to a specific HPV RNA probe and RNA-DNA hybrids were captured and detected by microplate chemiluminescence. Light signals were measured as relative light units (RLUs) with light intensity indicating the presence or absence of target DNA in the tested specimen. RLU measurements equal to or higher than the cutoff (CO) value (RLU/CO = 1) indicated the presence of HPV DNA in the sample. RLU/CO < 1 indicated the absence of specific HPV DNA or HPV DNA below the detection limit [13].

### 2.3. Tissue Microarray (TMA) Construction

For immunohistochemical analyses of BCL2, p21, p53, Rb, and survivin, tissue microarrays (TMAs) were constructed. Briefly, on H&E-stained slides of tumors, a representative area was selected and a corresponding spot was marked on the surface of the paraffin block. Using a biopsy needle, the selected area was punched out and a 1.5-mm tissue core was placed into a recipient block of 6 rows × 7 columns. The aforementioned selected tissues based on H&E-stained slides were then extracted. Two tissue cores were extracted from one patient's paraffin block to minimize extraction bias. Sections (4 *μ*m) from each TMA were stained with H&E to verify the presence of breast cancer tissues and were used for subsequent immunohistochemistry (IHC) analysis.

### 2.4. Immunohistochemistry (IHC)

IHC for BCL2, p21, p53, Rb, and survivin was performed using the streptavidin-peroxidase-biotin (SP) method [14, 15]. The sections were incubated with antibodies against BCL2, p21, p53, Rb, and survivin, respectively. BCL2, p21, p53, and survivin monoclonal antibodies were purchased from ZSGB-BIO (Beijing, China). Rb monoclonal antibody was obtained from Maixin Biotech (Fuzhou, China). Negative controls included substitution of the monoclonal antibody with PBS.

Immunohistochemical evaluation of all specimens was performed in a blinded manner. Staining for BCL2 and survivin was detected in the cell membrane/cytoplasm and cytoplasm/nucleus, respectively. The percentage of the BCL2 or survivin positive cells was scored as follows: 0, 0% positive cells; +1, <5%; +2, 5–20%; +3, 21–50%; +4, 51–75%; and +5, >75%. The intensity of the cellular staining was scored as follows: 0, absent; +1, weak; +2, moderate; +3, strong staining [16]. Both scores (percentage score and intensity score) were then multiplied, and scores ranging from 0 to 5 were considered as low expression, 6–10 moderate expression, and 11–15 high expression. For p53 and p21, cells which presented brown-yellow staining in the cell nuclei were considered to be positive as previously described [17]. For Rb, only nuclear labeling was analyzed. Cases were considered negative for RB when no neoplastic cell nuclei showed labeling in sections in which stromal cells and endothelial cells stained [18, 19].

### 2.5. Statistical Analysis

Data were analyzed using SPSS 20.0 software (SPSS Inc., Chicago, IL, USA). Statistical significance for categorical variables was determined by chi-squared and Fisher's exact tests. A *P* value < 0.05 was considered statistically significant.

## 3. Results

### 3.1. Patient Information and Clinicopathological Features

The study population consisted of 81 patients with breast cancer (Table 1). The median age of patients was 52 years (range, 31 to 78 years). The most abundant type of breast carcinoma was invasive ductal carcinoma (IDC) (79.0%, 64/81). Additionally, the cases include 11 ductal carcinomas in situ (DCIS), 2 invasive lobular carcinomas, 3 mucinous adenocarcinomas, and 1 papillary carcinoma. The median size of tumor was 2 cm (range: 0.5 to 6.5 cm), and 65.4% (53/81) of the patients were lymph node metastasis-negative. The estrogen receptor (ER), progesterone receptor (PR), and HER2-positive status was seen in 77.8%, 70.4%, and 22.4% of the patients, respectively.

### 3.2. HPV Status Demonstrated No Correlation with the Clinicopathological Parameters of Breast Cancer Patients

**Figure 1:**
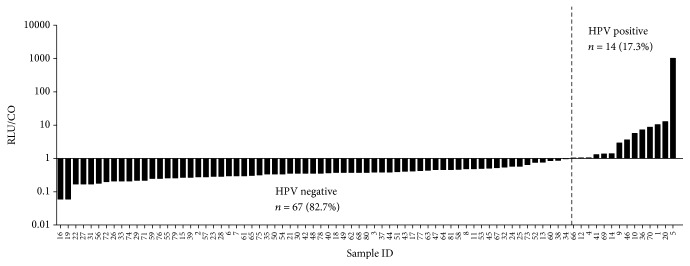
*Detection of HR-HPV in 81 fresh breast cancer samples using HC2 assay*. HPV signals were measured as relative light units (RLUs) with light intensity indicating the presence or absence of target DNA in the tested specimen. RLU measurements equal to or higher than the cutoff (CO) value (RLU/CO ≥ 1) indicated the presence of HPV DNA in the sample. RLU = CO indicates approximately 5,000 virus copies in the specimen. The prevalence of high-risk HPVs detected by HC2 was 17.3% (14/81) in breast cancer samples.

The prevalence of 13 types of high-risk HPV detected by HC2 was 17.3% (14/81) in 81 cases of breast cancer samples (Figure 1). Among the 14 positive cases, 12 were invasive ductal carcinoma and 2 were ductal carcinomas in situ. As shown in Table 1, no significant difference was observed in age (*P* = 0.140), histological type (*P* = 0.508), histological grade (*P* = 0.490), tumor size (*P* = 0.770), lymph node metastasis (*P* = 0.761), status of ER (*P* = 0.724), PR (*P* = 0.793), HER2 (*P* = 1.000), and Ki-67 expression (*P* = 0.554) between HPV-positive and HPV-negative cancers.

### 3.3. Association between HPV Status and BCL2, p21, p53, Rb, and Survivin Expression

**Figure 2:**
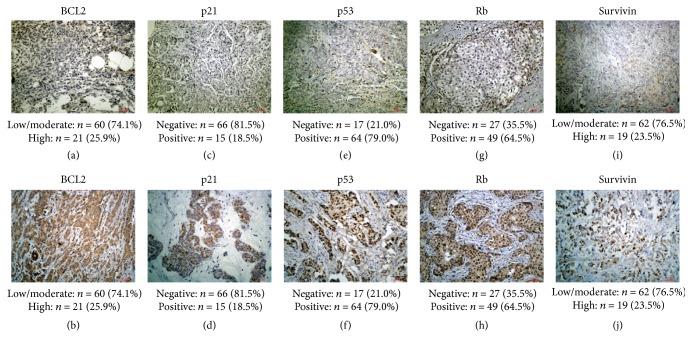
*Representative images of IHC assays analyzing the expression of BCL2, p21, p53, Rb, and survivin in breast cancer tissues*. BCL2 mainly showed cytoplasmic staining (a-b). Positive staining of P21 (c-d), p53 (e-f), and Rb (g-h) was observed in cell nuclei. Survivin staining (i-j) was seen in the cytoplasm and/or nucleus. High expression of BCL2 and survivin was seen in 26.0% and 23.5% of the cases, respectively. P21 positive expression was observed in 18.5% of the cases, p53 in 79.0% of the cases, and Rb in 64.5% of the cases.

Immunohistochemically, BCL2 mostly displayed cytoplasmic staining (Figures 2(a) and 2(b)). Positive staining of p21 (Figures 2(c) and 2(d)), p53 (Figures 2(e) and 2(f)), and Rb (Figures 2(g) and 2(h)) was mainly observed in nuclei of cancer cells. Positive survivin staining (Figures 2(i) and 2(j)) was seen in the cytoplasm and/or nucleus. Of all the cases, high expression of BCL2 and survivin was seen in 26.0% and 23.5% of the cases, respectively. P21 positive expression was observed in 18.5% of the cases, p53 in 79.0% of the cases, and Rb in 64.5% of the cases.

In addition, HR-HPV-positive tissues showed significantly higher expression of BCL2 (*P* = 0.041) and lower p53 (*P* = 0.008), compared with HPV-negative tissues (Table 2). Specifically, BCL2 was highly expressed in 7/14 (50.0%) of HPV-positive and 14/67 (20.9%) of HPV-negative breast cancers. P53 negativity was seen in 7/14 (50.0%) of HPV-positive and 10/67 (14.9%) of HPV-negative breast cancers. However, no significant differences were observed in p21, Rb, and survivin expression between HR-HPV-positive tissues and HR-HPV-negative tissues.

## 4. Discussion

Previous studies have shown that the prevalence of HPV in breast cancer worldwide ranges from 0 to 86% [20–23]. Potential explanations include (i) difficulties in detection due to low viral load and low frequency of HPV in breast cancers in some populations; (ii) differences between fresh samples and paraffin-embedded specimens; (iii) differences in detection methods; (iv) different histological types of breast tumors. In this study, high-risk HPVs were detected in 14 (17.3%) of 81 fresh breast cancer specimens, which is in accordance with a systematic review study conducted on European (13.4%) and America Central and South American (15.1%) [24]. However, higher HPV prevalence (42.9%) was found in breast cancer patients in North America and Australia [24]. Most recently, Haghshenas et al. reported that prevalence of HPV infection among Iranian women with breast cancer was 23.6% [25]. We comment that demographic features or ethnic factors may contribute to geographic differences of HPV infection. Notably, fresh breast cancer samples were used in this study and HC2 could detect 13 types of HR-HPV at the same time, which may improve the accuracy and HPV prevalence, compared to those utilizing paraffin-embedded specimens [4, 26]. Our present study, together with others, provides evidence that HPV may play a role in the development of breast cancers [27], although data linking breast cancer with HPV infection are contradictory.

Our results demonstrated no correlations of HPV status with any of the clinical features. Consistently, previous researchers also showed no significant correlation between HPV infection and the clinicopathological characteristics [13, 21]. However, Antonsson et al. found that HPV-positive breast cancers were smaller tumors with earlier T stage compared to HPV-negative cancers in a recent study covering 54 fresh frozen breast cancers [26]. Kroupis et al. reported that HPV-positive breast cancer patients were younger and with less ER positive rate and more proliferative index in 107 frozen breast cancer specimens [28].

Immunohistochemically detectable p53 is generally considered as protein product of genetic mutation [29]. However, several lines of studies have suggested that positive immunohistochemistry for p53 protein is not always indicative of p53 mutation [30, 31]. P53 alterations (mutation and/or nuclear protein accumulation determined by IHC) have been correlated with aggressive clinicopathological parameters and poor prognosis in breast carcinoma [29]. The positive rate of p53 in breast cancers has been reported to be 17% to 68% [17], and a slightly higher positivity (79%) was seen in our study. This discrepancy may be due to histological tumor type, tumor stage, antibodies, detection techniques, and evaluation methods. Here we showed less detectable p53 protein immunoreactivity in the HPV-positive breast carcinomas (50.0%) than the HPV-negative ones (85.1%). The observation supports the hypothesis of inactivation and degradation of p53 by HPV E6 in the HPV-positive breast carcinomas, and that p53 mutation is not necessary for transformation of these cases [17]. Inverse correlation between p53 expression and HPV infection has been documented in esophageal squamous cell carcinoma [32], endocervical adenocarcinoma [33], and breast cancer [17]. Our findings were in line with these studies.

The BCL2 protein is a member of the BCL family that inhibits apoptosis. Its oncogenic potential has been demonstrated in animal models [34] and a variety of tumors [35]. A recent report established its role as an independent prognostic biomarker as assessed by immunohistochemistry in breast cancer [36]. In the present study, we observed positive correlation between HPV infection and higher BCL2 expression in breast cancers, with more detectable BCL2 protein in HPV-positive than HPV-negative breast carcinomas (50.0% versus 20.9%). Other studies have also demonstrated a positive association between the presence of HR-HPV and BCL2 in breast cancer [37] and cervical cancer [38]. Since p53 and BCL2 are antagonistic in their function, an inverse correlation between their protein expression levels has been reported in many cancers [39, 40]. We found that HPV-positive tumors had significantly higher BCL2 and lower p53 expression than HPV-negative tumors, which is consistent with the hypothesis that the p53 protein inactivated by HR-HPV E6 releases the repression of BCL2 gene which leads to the overexpression of BCL2 protein [41], although it is not clear whether the upregulation of the BCL2 protein is a direct effect of HPV infection or an indirect effect through p53 protein inactivation in breast cancers.

No association was observed between HPV status and expression of p21, Rb, and survivin protein, suggesting that p21/Rb/survivin may not play major roles in HPV-associated carcinogenesis in these cases.

A major limitation of this study was the relatively small sample size, resulting in a loss of statistical power for some analyses. Thus, we could not rule out the possible involvement of p21, Rb, and survivin in HPV infection and breast carcinogenesis. Another potential limitation was the lack of survival analysis, failing to assess the prognostic value of HPV infection and the cell cycle/apoptosis regulatory proteins. Further studies with larger samples and follow-up data are deserved for confirmation of the current findings.

In conclusion, our study did provide evidence of high-risk HPV prevalence in breast cancer. Additionally, our findings suggest significant correlations between HPV infection and p53 and BCL2 expression. Further studies are necessary to investigate the underlying mechanisms through which HPV causes dysregulation of p53 and BCL2 and their roles as prognostic markers in breast cancer.

## Figures and Tables

**Table 1:** Relationship between HPV and clinicopathological parameters in breast cancers.

Features	*n*	HPV		*P*
		Negative	Positive	
*Age (y)*				
≤50	45	40	5	
>50	36	27	9	0.140
*Histological type*				
DCIS	11	9	2	
IDC	64	52	12	
Others	6	6	0	0.508
*Grade*				
II	47	37	10	
III	17	15	2	0.490
Unknown	17			
*Tumor size (cm)*				
≤2	43	36	7	
>2	35	28	7	0.770
Unknown	3			
*Lymph node metastasis*				
Negative	53	43	10	
Positive	28	24	4	0.761
*ER*				
Negative	18	16	2	
Positive	63	51	12	0.724
*PR*				
Negative	24	20	4	
Positive	57	47	10	0.793
*HER2*				
Negative	52	44	8	
Positive	15	13	2	1.000
Unknown	14			
*Ki-67*				
Negative	29	23	6	
Positive	52	44	8	0.554

DCIS: ductal carcinomas in situ; IDC: invasive ductal carcinoma; Others: invasive lobular carcinomas, mucinous adenocarcinomas, and papillary carcinoma.

**Table 2:** Relationship between HPV status and BCL2, p21, p53, Rb, and survivin expression in breast cancers.

Features	HPV		*P*
	Negative	Positive	
*BCL2*			
Low/moderate	53	7	
High	14	7	0.041
*p21*			
Negative	56	10	
Positive	11	4	0.280
*p53*			
Negative	10	7	
Positive	57	7	0.008
*Rb* ^1^			
Negative	24	3	
Positive	39	10	0.359
*Survivin*			
Low/moderate	51	11	
High	16	3	1.000

^1^Seventy-six cases were available for Rb IHC assessment.
